# Real-time polymerase chain reaction methods for strain specific identification and enumeration of strain *Lacticaseibacillus paracasei* 8700:2

**DOI:** 10.3389/fmicb.2022.1076631

**Published:** 2023-01-18

**Authors:** Hanan R. Shehata, Basma Hassane, Steven G. Newmaster

**Affiliations:** ^1^Natural Health Product Research Alliance, Department of Integrative Biology, College of Biological Science, University of Guelph, Guelph, ON, Canada; ^2^Department of Microbiology, Faculty of Pharmacy, Mansoura University, Mansoura, Egypt; ^3^Purity-IQ Inc., Guelph, ON, Canada

**Keywords:** real-time PCR, probe-based assay, strain specific PCR assay, probiotics, viability PCR, PMAxx, *Lactobacillus paracasei* 8700:2, viable but non-culturable

## Abstract

**Introduction:**

Reliable and accurate methods for probiotic identification and enumeration, at the strain level plays a major role in confirming product efficacy since probiotic health benefits are strain-specific and dose-dependent. In this study, real-time PCR methods were developed for strain specific identification and enumeration of *L. paracasei* 8700:2, a probiotic strain that plays a role in fighting the common cold.

**Methods:**

The assay was designed to target a unique region in *L. paracasei* 8700:2 genome sequence to achieve strain level specificity. The identification assay was evaluated for specificity and sensitivity. The enumeration viability real-time PCR (v-qPCR) method was first optimized for the viability treatment, then the method was evaluated for efficiency, limit of quantification, precision, and its performance was compared to plate count (PC) and viability droplet digital PCR (v-ddPCR) methods.

**Results:**

The identification method proved to be strain specific and highly sensitive with a limit of detection of 0.5 pg of DNA. The optimal viability dye (PMAxx) concentration was 50 μM. The method was efficient (> 90% with *R*^2^ values > 0.99), with a linear dynamic range between 6*10^2^ and 6*10^5^ copies. The method was highly precise with a relative standard deviation below 5%. The Pearson correlation coefficient (*r*) was 0.707 for PC and v-qPCR methods, and 0.922 for v-qPCR and v-ddPCR. Bland-Altman method comparison showed that v-qPCR always gave higher values compared to PC method (relative difference ranging from 119% to 184%) and showed no consistent trend (relative difference ranging from −20% to 22%) when comparing v-qPCR and v-ddPCR methods.

**Discussion:**

The difference between PC and v-PCR methods can potentially be attributed to the proportion of cells that exist in a viable but non culturable (VBNC) state, which can be count by v-PCR but not with PC. The developed v-qPCR method was confirmed to be strain specific, sensitive, efficient, with low variance, able to count VBNC cells, and has shorter time to results compared to plate count methods. Thus, the identification and enumeration methods developed for *L. paracasei* 8700:2 will be of great importance to achieve high quality and efficacious probiotic products.

## Introduction

Public awareness of probiotic health benefits is expanding, and probiotic consumption is rapidly increasing, with a global market size valued at USD 58.17 billion in 2021. The global market size is anticipated to continue to grow at a compound annual growth rate of 7.5% to reach USD 111.21 billion in 2030 (Grand-View-Research-Inc, 2022). Probiotics are sold as dietary supplements in multiple finished forms such as capsules, tablets, powders, oils, and gummies, and are also incorporated in several beverages and foods products. Probiotic health benefits are diverse; however, health benefits are strain-specific (Klein et al., 2010; Sánchez et al., 2017; McFarland et al., 2018). This makes it essential for probiotic products to contain the correct strains to achieve their claimed health benefits. Additionally, health benefits are dose-dependent, so it is equally important for a product to contain the correct dose throughout shelf life to achieve product efficacy (Tripathi and Giri, 2014; Kolaček et al., 2017; Sánchez et al., 2017). Thus, availability of reliable methods for probiotic identification and enumeration, at the strain level, plays a major role in producing efficacious products.

The most common methods for probiotic strain identification are DNA based methods, such as conventional PCR and real-time PCR methods that can be designed to achieve species level or strain level identification (Solano-Aguilar et al., 2008; Ahlroos and Tynkkynen, 2009; Achilleos and Berthier, 2013; Herbel et al., 2013; Morovic et al., 2016; Shehata et al., 2020, 2021a; Shehata and Newmaster, 2020; Zhang et al., 2021). Real-time PCR based methods have several advantages such as the ability to monitor reactions in real time, high sensitivity, high accuracy, and short time to results. In addition, these methods eliminate post-PCR processing, which minimizes the risk of cross contamination (Wilhelm and Pingoud, 2003).

Quantification or enumeration of probiotics can be achieved using several orthogonal methods. The plate count method is the standard method for viable count determination of probiotics (Hill and Slack, 1904; Hill, 1908; Breed and Dotterrer, 1916; Davis, 2014), including ISO approved methods for the enumeration of *Lactobacillus acidophilus* and Bifidobacteria (ISO, 2006, 2010). Plate count methods, however, do not enable strain specific enumeration and typically require incubation for 2 or more days for colonies to grow. These methods are also laborious, costly and highly variable (Hansen et al., 2018; Jackson et al., 2019). Moreover, plate count methods are unable to count cells that are in a viable but non culturable (VBNC) state as these methods measure cultivability (Fiore et al., 2020). Another method for viable count determination is flow cytometry (ISO, 2015). This method relies on membrane integrity as an indicator for cell viability. Thus, it enables the enumeration of VBNC cells (Hansen et al., 2020). Flow cytometry does not allow taxa specific enumeration unless taxa specific antibodies are used (Chiron et al., 2018). Given the strain specificity of probiotic health benefits, viable count determination methods that are strain specific are needed. A third method for viable count determination is DNA based methods such as real-time PCR and digital PCR methods. These methods can enable strain specific enumeration (García-Cayuela et al., 2009; Kramer et al., 2009), since they can detect small genetic variations between closely related strains such as single nucleotide polymorphisms, by using strain specific primers and probes. In addition to strain specificity, PCR based methods offer high sensitivity and accuracy and faster time to results. To use these methods for the enumeration of viable cells only, cells should be pre-treated with viability dyes such as ethidium monoazide (EMA) and propidium monoazide (PMA). Viability dyes can enter dead cells and membrane damaged cells only, intercalating to their DNA, making it unreactive in PCR (Fittipaldi et al., 2012; Shehata and Newmaster, 2021). Thus, these viability-PCR methods will enable enumeration of viable cells only. Viability-PCR methods can count VBNC cells, which cannot be captured by plate count methods although they maintain probiotic properties (Davis, 2014).

*Lacticaseibacillus paracasei* 8700:2, formerly, *L. paracasei* 8700:2 (DSM 13434) is a probiotic strain that was originally isolated from human gut and its genome sequence was deposited in GenBank under accession number CP002391.1. The health benefits of the strain were studied in several *in vitro* studies and in clinical trials. For example, the daily consumption of 10^9^ CFU (colony forming units) of the combination of *L. plantarum* HEAL9 and *L. paracasei* 8700:2 (Probi Defendum) for three months significantly reduced the severity of common cold symptoms “nasal congestion and runny nose” in healthy children 1–6 years of age in a randomized, double-blind, placebo-controlled clinical study (Lazou Ahrén et al., 2020). Intake of the probiotic strains *L. plantarum* HEAL 9 and *L. paracasei* 8700:2 was reported to reduce the risk of acquiring common cold infections or reduce common cold symptoms and duration in randomized, double-blind and placebo-controlled studies (Berggren et al., 2011; Regina et al., 2013). Additionally, *L. paracasei* 8700:2 showed *in vitro* antagonistic activities against *Salmonella enterica* ssp. *enterica* and *Helicobacter pylori* (Hütt et al., 2006). *L. paracasei* 8700:2 used in combination with *L. plantarum* HEAL9 was also shown to decrease attack frequency in periodic fever, aphthous stomatitis, pharyngitis, and adenitis (PFAPA) syndrome patients (Batu et al., 2022). In this study, real-time PCR methods were developed and validated to enable strain specific identification and viable count determination of strain *L. paracasei* 8700:2. The methods can be employed for quality control purposes and for strain tracking in clinical trials.

## Materials and methods

### Reference materials and DNA extraction

A total of 10 reference samples of *Lacticaseibacillus paracasei* 8700:2 was acquired from The Bountiful Company (United States). Five samples were mono-strain samples, and five samples were multi-strain samples (Table 1). Additionally, reference materials from 23 non-target probiotic strains were obtained from International Flavors and Fragrances (previously DuPont Nutrition & Biosciences), Nature’s Way Brands, The Bountiful Company, Lallemand Health Solutions and Chr. Hansen (UAS Labs; Table 1). DNA was extracted and quantified as previously described (Shehata et al., 2021b).

**Table 1:** Samples used to evaluate the analytical specificity of *Lacticaseibacillus paracasei* 8700:2 strain-specific assay and results for analytical specificity testing.

Sample ID	Sample type	Strain	Mean Cq + SEM^#^
1	Target (Mono-strain)	*L. paracasei* 8700:2	23.98 ± 0.025
2	Target (Mono-strain)	*L. paracasei* 8700:2	24.24 ± 0.024
3	Target (Mono-strain)	*L. paracasei* 8700:2	24.14 ± 0.021
4	Target (Mono-strain)	*L. paracasei* 8700:2	24.09 ± 0.019
5	Target (Mono-strain)	*L. paracasei* 8700:2	24.21 ± 0.061
6	Target (Multi-strain)	*L. paracasei* 8700:2 with other strains	24.69 ± 0.018
7	Target (Multi-strain)	*L. paracasei* 8700:2 with other strains	24.34 ± 0.052
8	Target (Multi-strain)	*L. paracasei* 8700:2 with other strains	24.16 ± 0.021
9	Target (Multi-strain)	*L. paracasei* 8700:2 with other strains	24.68 ± 0.019
10	Target (Multi-strain)	*L. paracasei* 8700:2 with other strains	24.86 ± 0.030
11	Non-target	*Lacticaseibacillus paracasei* HA-196	NA^*^
12	Non-target	*Lacticaseibacillus paracasei* HA-274	NA
13	Non-target	*Lacticaseibacillus paracasei* R0215	NA
14	Non-target	*Lacticaseibacillus paracasei* R0422	NA
15	Non-target	*Lacticaseibacillus paracasei* HA-108	NA
16	Non-target	*Lacticaseibacillus paracasei* UALpc-04	NA
17	Non-target	*Lacticaseibacillus paracasei* Lpc-37	NA
18	Non-target	*Lacticaseibacillus casei* UALc-03	NA
19	Non-target	*Lacticaseibacillus casei* Lc-11	NA
20	Non-target	*Lacticaseibacillus rhamnosus* GG	NA
21	Non-target	*Lacticaseibacillus rhamnosus* Lr-32	NA
22	Non-target	*Lactobacillus acidophilus* La-14	NA
23	Non-target	*Lactobacillus acidophilus* NCFM	NA
24	Non-target	*Lactiplantibacillus plantarum* Lp-115	NA
25	Non-target	*Levilactobacillus brevis* Lbr-35	NA
26	Non-target	*Ligilactobacillus salivarius* Ls-33	NA
27	Non-target	*Lactobacillus gasseri* Lg-36	NA
28	Non-target	*Limosilactobacillus reuteri* 1E1	NA
29	Non-target	*Bifidobacterium animalis* subsp. lactis Bl-04	NA
30	Non-target	*Bifidobacterium longum* subsp. infantis Bi-26	NA
31	Non-target	*Bifidobacterium longum* Bl-05	NA
32	Non-target	*Bifidobacterium bifidum* Bb-06	NA
33	Non-target	*Bifidobacterium breve* Bb-03	NA

^#^SEM, standard error of mean. ^*^NA means no amplification.

### Real-time PCR assay design

To design a strain-specific real-time PCR assay for strain *L. paracasei* 8700:2, Rapid Annotation using Subsystem Technology (RAST) was used to identify a unique sequence region in the genome of strain *L. paracasei* 8700:2 (GenBank: CP002391.1) compared to closely related *L. paracasei* strains (Aziz et al., 2008; Overbeek et al., 2014; Brettin et al., 2015). The identified sequence region was used to design primers and a hydrolysis probe, using PrimerQuest Tool [Integrated DNA Technologies (IDT), Coralville, IA, United States] and were ordered from IDT (Table 2).

**Table 2:** *Lacticaseibacillus paracasei* 8700:2 strain specific primer and probe sequences.

Primer F	5'-GGAACTCGTAGCATCTACTAAGC-3'
Primer R	5'-CTATGGCCTTGTCTCCTTCTTC-3'
Probe	5'-AGAATCCACAAGAGACGCCCAAAGT-3' (56-FAM and ZEN—3IABkFQ)

### Real-time PCR protocol

Primer and probe working solutions were prepared at 10 and 5 μM, respectively. Each PCR reaction (20 μl total volume) consisted of 10 μl of 2 × SensiFast Probes Master Mix (BIO-86020, Bioline), 4.4 μl of molecular biology grade water, 1.8 μl of each primer (10 μM), 1.0 μl of probe (5 μM), and 1 μl of DNA. PCR running protocol is as follows: denaturation at 95°C for 5 min and amplification (95°C for 10 s, and 60°C for 20 s) for 40 cycles. No template controls (NTC) were included in each run. All samples were tested in triplicate. All tests were conducted on Hyris bcube platform and Cq values were calculated using Hyris bApp.

### Evaluating the analytical specificity of *Lacticaseibacillus paracasei* 8700:2 strain-specific identification method

The specificity of the developed method was first evaluated *in silico*. The identified target region was searched on NCBI GenBank using the Basic Local Alignment Search Tool (BLAST) nucleotide function against all publicly available sequences to confirm the uniqueness of the identified target region to strain *L. paracasei* 8700:2. The specificity of the developed method was also evaluated experimentally by testing 10 target samples and 23 non-target probiotic strains (Table 1). The panel of non-target strains included other *L. paracasei* and *L. casei* strains (HA-196, HA-274, R0215, R0422, HA-108, UALpc-04, Lpc-37, UALc-03, and Lc-11) to confirm strain level specificity (Shehata et al., 2019). DNA from all samples was diluted to 1 ng/μl before use in the real-time PCR protocol described above. True positive rates and false positive rates were calculated (Codex-Alimentarius-Commission, 2010; Shehata et al., 2019).

### Evaluating the analytical sensitivity of *Lacticaseibacillus paracasei* 8700:2 strain-specific identification method

To determine assay sensitivity or limit of detection (LOD), three series of DNA dilutions starting from 10, 5, and 2 ng/μl were used (Shehata et al., 2019; Shehata and Newmaster, 2020). Each dilution series consisted of 5 dilution points (Bustin et al., 2009).

### Optimization of viability treatment of *Lacticaseibacillus paracasei* 8700:2 strain-specific enumeration method

To use the strain specific primers and probe for viable count determination, the assay needs to be coupled with a viability dye treatment (Gobert et al., 2018). To find the optimal concentration of viability dye that can effectively inactivate DNA from dead cells, heat-killed and non-heated cells from one mono-strain reference sample were treated with the viability dye PMAxx (40069, Biotium Inc., Hayward, CA, United States) at final concentrations of 0, 25, 50, 100, and 150 μM as previously described (Shehata and Newmaster, 2021). Once a potential optimal PMAxx concentration was determined, it was confirmed using five mono-strain reference samples. To liberate DNA following PMAxx treatments, bead beating in BeadBug™ prefilled tubes (Z763764, Sigma-Aldrich, St. Louis, MO, United States) was performed for 5 min at 3,000 rpm (Hansen et al., 2018). The liberated DNA was used in real-time PCR.

### Determination of linear dynamic range, and reaction efficiency of *Lacticaseibacillus paracasei* 8700:2 strain-specific enumeration method

To determine the limit of quantification (LOQ), linear dynamic range and efficiency of this assay, 10-fold serial dilutions were prepared from three *L. paracasei* 8700:2 reference samples at five dilution points each as previously described (Shehata and Newmaster, 2021). A calibration curve was then established between Cq and log_10_ genome number/ml using Prism 9 (GraphPad Software, San Diego, CA, United States). Reaction efficiency values were calculated from the slopes.

### Evaluating precision of *Lacticaseibacillus paracasei* 8700:2 strain-specific enumeration method

The assay was evaluated for repeatability and reproducibility by repeating the method using two samples which were tested at four different concentrations each (10^−3^, 10^−4^, 10^−5^, and 10^−6^). To determine repeatability, the method was repeated over a short period of time, while for reproducibility, the method was repeated on multiple bCUBE machines. The variation was calculated as the relative standard deviation (RSD%).

### Comparing *Lacticaseibacillus paracasei* 8700:2 viability real-time PCR enumeration method to plate count and viability droplet digital PCR methods

The viable counts of *L. paracasei* 8700:2 in 10 samples (5 mono-strain and 5 multi-strain experimental mixtures) were determined using the developed viability real-time PCR method (v-qPCR). Viable counts were also determined using plate count method (PC) as previously described (Shehata and Newmaster, 2021). The plate count of *L. paracasei* 8700:2 in multi-strain experimental mixtures was determined before blending mono-strain samples of *L. paracasei* 8700:2 in the experimental mixtures.

The viable counts of *L. paracasei* 8700:2 in the ten samples were also determined using viability droplet digital PCR (v-ddPCR). The ddPCR reaction mixture consisted of 1 × ddPCR Supermix for Probe (Bio-Rad, Mississauga, ON), 48 nM each of the primers and 48 nM probe, and 5 μl of sample DNA in a final volume of 25 μl. In a DG8 Cartridge (Bio-Rad), 20 μl from each reaction mixture were mixed with 70 μl of Droplet Generation oil for Probes (Bio-Rad). PCR droplets were then generated using a QX200^™^ Droplet Generator (Bio-Rad). From each droplet mix, 20 μl were transferred to a 96-well PCR plate (Bio-Rad). The plate was sealed with a foil heat seal using a PX1^™^ PCR plate Sealer (Bio-Rad). PCR amplification was carried out on a GeneAmp™ PCR System 9700 (Applied Biosystems, Foster City, CA, United States) under the following settings: 95°C for 10 min, followed by 48 cycles of 95°C for 20 s and 60°C for 40 s, and 1 cycle of 98°C for 10 min. After amplification, droplets from each well were read automatically using a QX200^™^ droplet reader (Bio-Rad). ddPCR data were acquired and analyzed with QuantaSoft software (Bio-Rad) and recorded as copies/μl.

### Assessing the effect of heat treatments on viable counts determined using *Lacticaseibacillus paracasei* 8700:2 viability real-time PCR and plate count methods

To compare the performance of viability real-time PCR and plate count methods in determining viable counts of heat-treated cells, fractions of a suspension of a mono-strain sample of *L. paracasei* 8700:2 were heated at 40, 50, 60, 70, 80, and 95°C. All heat-treated cells were treated with PMAxx at a final concentration of 50 μM followed by bead beating as described above. Viable counts were determined using viability real-time PCR and plate count methods as described above.

### Assessing the ability of *Lacticaseibacillus paracasei* 8700:2 viability real-time PCR enumeration method to detect cell death following storage

To assess the ability of the developed method to detect cell death following storage, the viable counts in five mono-strain samples of *L. paracasei* 8700:2 were determined using viability real-time PCR and plate count methods at two different time points, 1 year apart. The samples were treated with a final PMAxx concentration of 50 μM followed by bead beating, followed by viable count determination using viability real-time PCR and plate count methods as described above.

### Statistical analysis

Prism 9 (GraphPad Software, San Diego, CA, United States) was used for statistical analyzes and preparing graphical displays.

## Results

### Real-time PCR assay design

Bioinformatic analysis using RAST identified a unique region in the genome of *L. paracasei* 8700:2. The region codes for a hypothetical protein (RAST and NCBI blastx). The designed primers and probe amplify a 149 bp amplicon (Table 2).

### Evaluating the analytical specificity of *Lacticaseibacillus paracasei* 8700:2 strain-specific identification method

*In silico* evaluation of assay specificity by searching the amplicon sequence on NCBI GenBank using BLAST revealed no similarity hits to any other bacteria (taxid:2, December 2021), which confirmed the uniqueness of the target sequence to *L. paracasei* 8700:2. Experimental evaluation of assay specificity by testing 10 target samples and 23 non-target probiotic strains showed that all target samples successfully amplified at mean Cq between 23.98 and 24.86. None of 23 non-target probiotic strains showed any amplification, including other *L. paracasei* and *L. casei* strains (HA-196, HA-274, R0215, R0422, HA-108, UALpc-04, Lpc-37, UALc-03, Lc-11; Table 1), indicating strain level specificity. True positive rate was 100% and false positive rate was 0%.

### Evaluating the analytical sensitivity of *Lacticaseibacillus paracasei* 8700:2 strain-specific identification method

**Figure 1:**
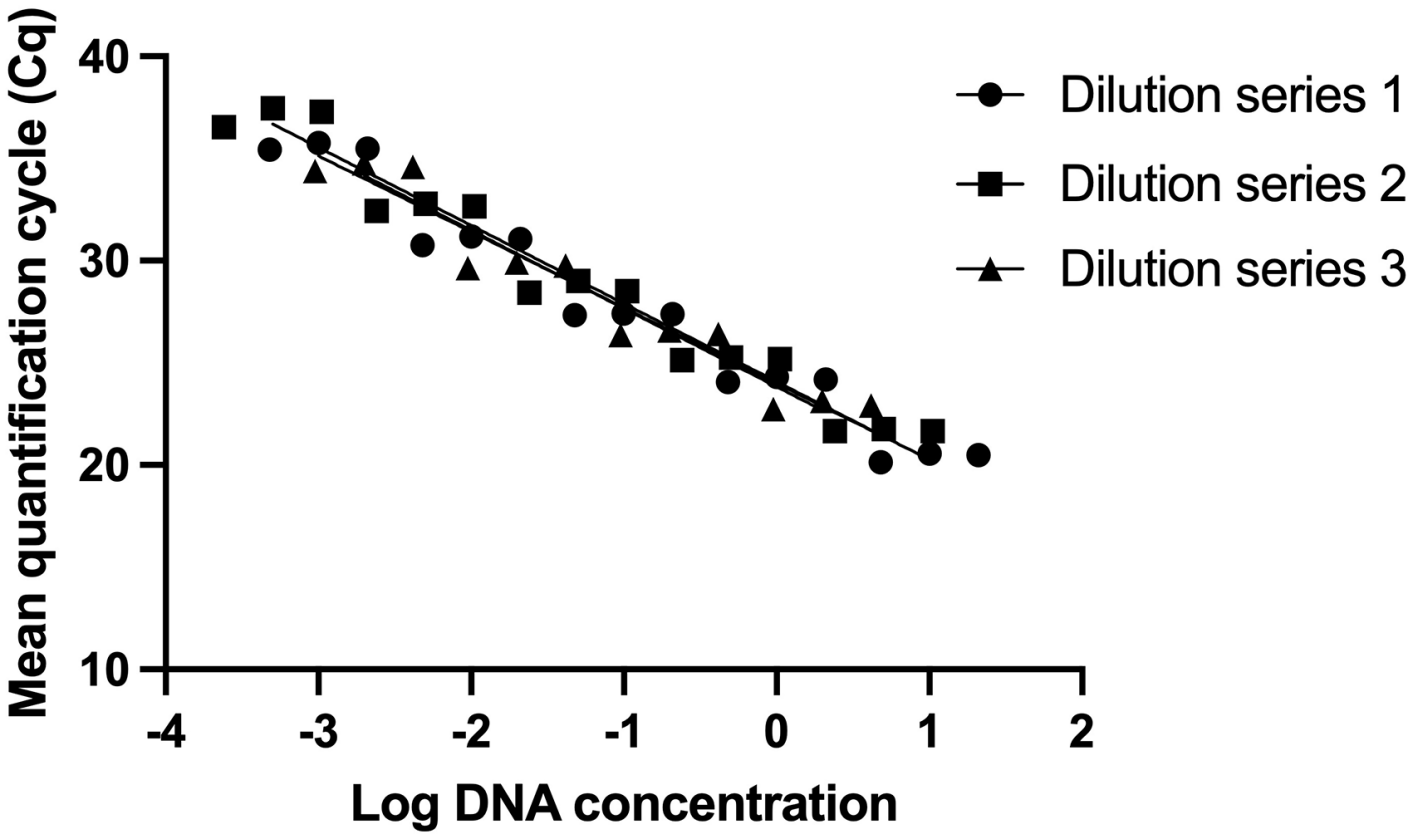
Evaluating the analytical sensitivity of *Lacticaseibacillus paracasei* 8700:2 strain-specific identification assay. Three DNA dilution series were prepared from starting concentrations of 10 ng/μl (Dilution series 1), 5 ng/μl (Dilution series 2), and 2 ng/μl (Dilution series 3). Dilution series consisted of five points each (10-fold) and each dilution was tested in triplicate to establish standard curves. The limit of detection was determined to be 0.5 pg. of DNA, corresponding to 150 target copies.

Standard curves were established from three DNA dilution series starting from 10, 5, and 2 ng/μl (Shehata et al., 2019; Shehata and Newmaster, 2020). The limit of detection was determined to be 0.5 pg. of DNA, corresponding to 150 target copies (Figure 1).

### Optimization of viability treatment of *Lacticaseibacillus paracasei* 8700:2 strain-specific enumeration method

**Figure 2:**
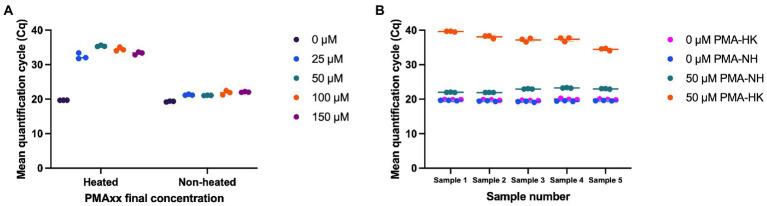
Optimization of viability treatment of *L. paracasei* 8700:2 strain-specific quantitative assay. **(A)** Final concentrations of PMAxx of 0, 25, 50, 100, and 150 μM were evaluated. PMAxx at concentrations of 25, 50, 100, and 150 μM resulted in significant shifts in Cq values from heat killed samples. PMAxx at a final concentration of 50 μM was selected as a potential optimal concentration. **(B)** Confirming 50 μM as an efficient PMAxx concentration using five mono-strain reference samples.

Multiple concentrations of PMAxx (0, 25, 50, 100, and 150 μM) were evaluated for effectiveness in inactivating DNA from dead cells. With no PMAxx, mean Cq value from non-heated cells and heat killed cells were 19.31 and 19.68, respectively. PMAxx at concentrations of 25, 50, 100, and 150 μM resulted in significant shifts in Cq values from heat killed samples (Mean Cq = 32.39, 35.41, 34.47, and 33.30, respectively), while treating non-heated cells with PMAxx at concentrations of 25, 50, 100, and 150 μM resulted in Cq values of 21.22, 21.06, 21.84, and 22.04, respectively (Figure 2A). PMAxx at a final concentration of 50 μM was selected as a potential optimal concentration. The optimal concentration was confirmed using five mono-strain reference samples. Using 50 μM final concentration of PMAxx resulted in significant shifts in Cq values from heat killed cells (Mean Cq ranged from 34.44 to 39.62 for the five samples; Figure 2B). PMAxx at a final concentration of 50 μM was selected as an efficient treatment for inactivating DNA from dead cells.

### Determination of linear dynamic range, and reaction efficiency of *Lacticaseibacillus paracasei* 8700:2 strain-specific enumeration method

**Figure 3:**
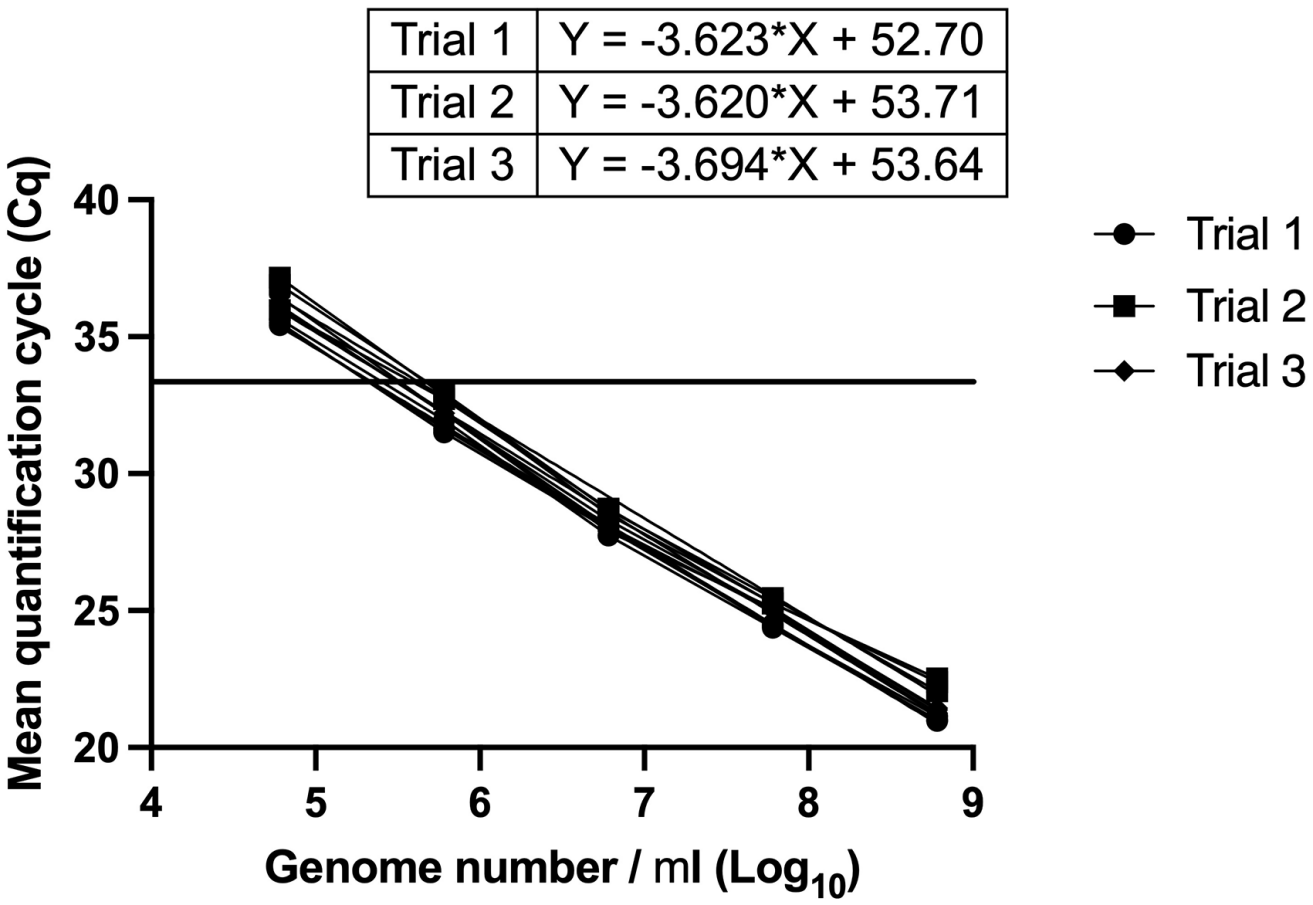
Determination of linear dynamic range, and reaction efficiency of *L. paracasei* 8700:2 strain-specific quantitative assay. Three independent serial dilution series were prepared from *L. paracasei* 8700:2 reference samples. The linear dynamic range was established between 6*10^2^ to 6*10^5^ genomes. Reaction efficiency was 91.5, 93.7, and 90.9%. A vertical line at a Cq value of 33 cycles refers to reaction cut-off.

The linear dynamic range was established between 6*10^2^ and 6*10^5^ genomes (Figure 3). With a cut-off value of 33 cycles, the Limit of Quantification was equivalent to 6*10^2^ genomes. The reaction efficiency was 91.5, 93.7 and 90.9% with *R*^2^ values = 0.9984, 0.9928 and 0.9965, and *p* value < 0.0001 in three independent trials (Figure 3). A cut-off value of 33 cycles was selected because reaction efficiency started to decline after 33 cycles.

### Evaluating precision of *Lacticaseibacillus paracasei* 8700:2 strain-specific enumeration method

**Figure 4:**
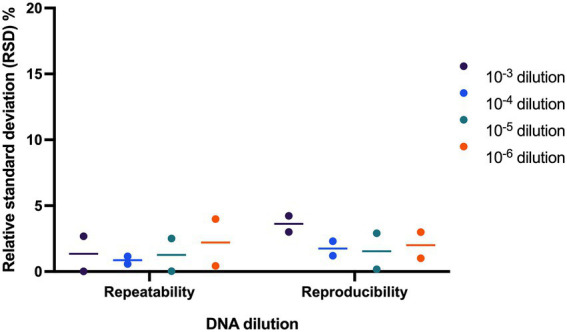
Evaluating precision of *L. paracasei* 8700:2 strain-specific quantitative assay. Two samples were tested at four dilutions to determine assay precision. The relative standard deviation (RSD%) ranged from 0.02 to 3.99% and from 0.18 to 4.23% for repeatability and reproducibility, respectively.

Repeatability and reproducibility of the assay were evaluated using two samples tested at four DNA dilution points. The RSD% ranged from 0.02 to 3.99% for repeatability and ranged from 0.18 to 4.23% for reproducibility (Figure 4).

### Comparing *Lacticaseibacillus paracasei* 8700:2 viability real-time PCR enumeration method to plate count and viability droplet digital PCR methods

**Figure 5:**
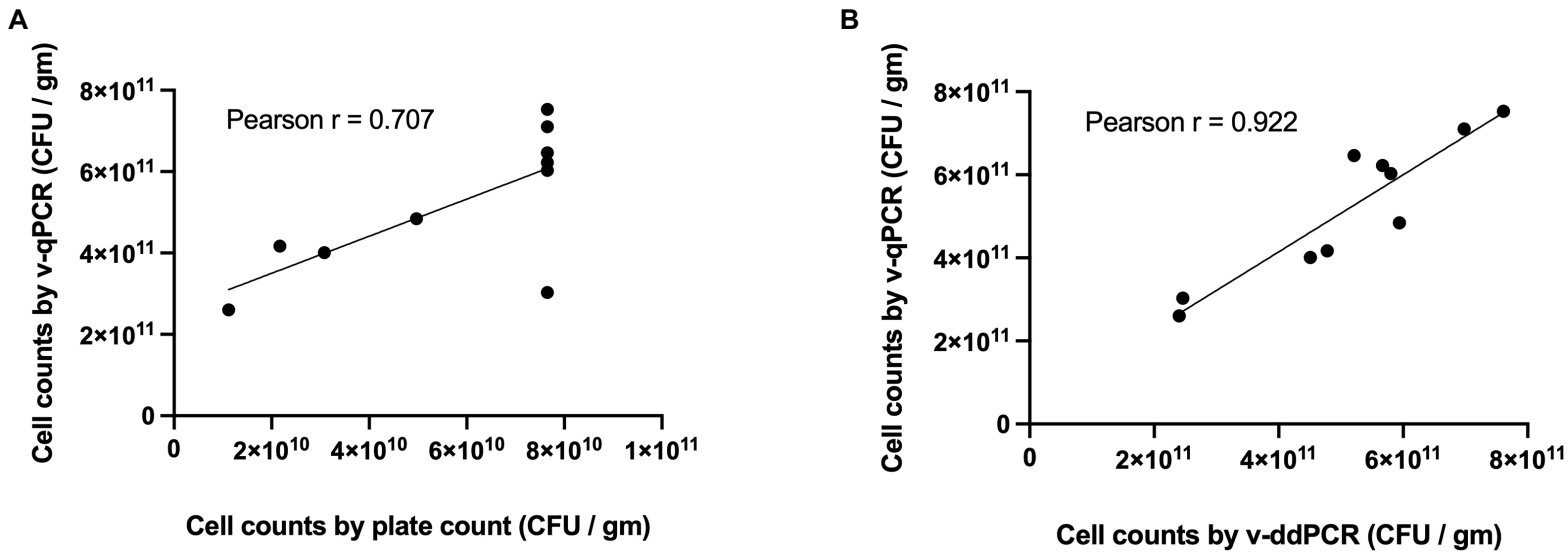
Correlation of *L. paracasei* 8700:2 viability real-time PCR method to plate count and viability droplet digital PCR methods. **(A)** Correlation between cell counts determined using viability real-time PCR (v-qPCR) and plate count (PC) method (Pearson *r* = 0.707). **(B)** Correlation between cell counts determined using viability real-time PCR (v-qPCR) and viability droplet digital PCR (v-ddPCR; Pearson *r* = 0.922).

The viable counts of *Lacticaseibacillus paracasei* 8700:2 in 10 samples (5 mono-strain and 5 multi-strain experimental mixtures) were determined using the developed v-qPCR, PC, and v-ddPCR. The Pearson correlation coefficient (*r*) was 0.707 for PC and v-qPCR methods (*p*-value = 0.0222; Figure 5A), and 0.922 for v-qPCR and v-ddPCR (*p*-value = 0.0001; Figure 5B).

**Figure 6:**
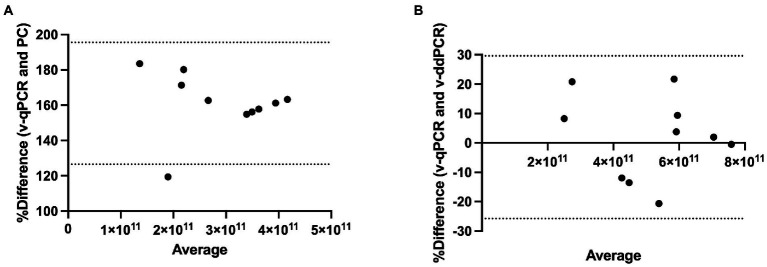
Method agreement analysis for *L. paracasei* 8700:2 viability real-time PCR method with plate count and viability droplet digital PCR methods. **(A)** Bland–Altman method comparison (difference versus average analysis) for viability real-time PCR (v-qPCR) method and plate count (PC) method (Relative difference ranging from 119 to 184%). **(B)** Bland–Altman method comparison (difference versus average analysis) for viability real-time PCR (v-qPCR) method and viability droplet digital PCR (v-ddPCR) method (Relative difference ranging from −20 to 22%).

Method agreement analysis was conducted using Bland–Altman method comparison (difference versus average analysis) for v-qPCR and PC methods as well as for v-qPCR and v-ddPCR methods. v-qPCR always gave higher values compared to PC method (Relative difference ranging from 119 to 184%; Figure 6A). The difference was significant (two-tailed *t*-test value of *p* < 0.0001). On the other hand, when comparing v-qPCR and v-ddPCR methods, there was no consistent trend (Relative difference ranging from −20 to 22%), and there was no significant difference (two-tailed *t*-test value of *p* = 0.7745; Figure 6B).

### Assessing the effect of heat treatments on viable counts determined using *Lacticaseibacillus paracasei* 8700:2 viability real-time PCR and plate count methods

Heating at 60, 70, 80, and 95°C resulted in no detectable viable cells using both viability real-time PCR and plate count methods. Heating at 50°C resulted in 9-fold reduction using plate count method and 5-fold reduction using viability real-time PCR compared to non-heated cells. Heating at 40°C resulted in 3-fold reduction using plate count method and 2-fold reduction using viability real-time PCR compared to non-heated cells (Table 3).

**Table 3:** Assessing the effect of heat treatments on viable counts determined using *L. paracasei* 8700:2 viability real-time PCR and plate count methods.

Heat treatment	Plate count method					Viability real-time PCR				
	Viable cell count			Mean	Fold reduction	Viable cell count			Mean	Fold reduction
Non-heated	7.5E+10	8.0E+10	7.6E+10	7.7E+10		5.9E+11	6.2E+11	6.6E+11	6.2E+11	
40°C	2.7E+10	2.6E+10	2.6E+10	2.6E+10	3	2.7E+11	3.1E+11	3.7E+11	3.2E+11	2
50°C	9.1E+09	8.0E+09	8.7E+09	8.6E+09	9	1.4E+11	1.4E+11	1.3E+11	1.4E+11	5
60°C	0	0	0	0		0	0	0	0	
70°C	0	0	0	0		0	0	0	0	
80°C	0	0	0	0		0	0	0	0	
95°C	0	0	0	0		0	0	0	0	

### Assessing the ability of *Lacticaseibacillus paracasei* 8700:2 viability real-time PCR enumeration method to detect cell death following storage

**Figure 7:**
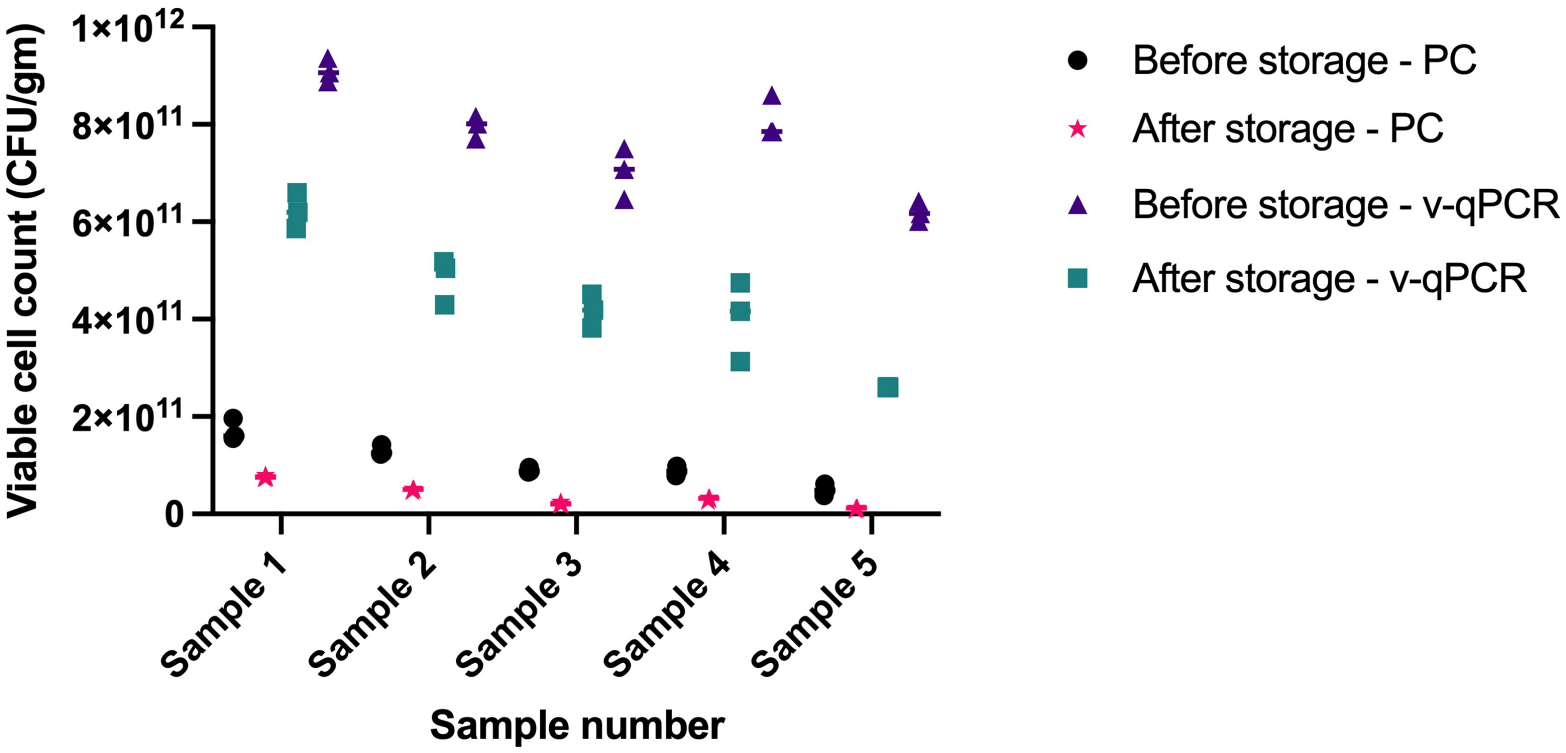
Assessing the ability of *L. paracasei* 8700:2 viability real-time PCR method to detect cell death following storage. Storage for 1 year resulted in 2.2-to-4.4-fold reduction in cell counts using plate count (PC) method and 1.5 to 2.4 fold reduction in cell counts using viability real-time PCR (v-qPCR).

The viable counts in five samples were determined using viability real-time PCR and plate count methods at two different time points, 1 year apart. Both methods were able to detect cell death over time. Storage for 1 year resulted in 2.2- to 4.4-fold reduction using plate count method and 1.5- to 2.4-fold reduction using viability real-time PCR (Figure 7).

## Discussion

Probiotic health benefits are strain specific and are dose-dependent. Thus, commercial probiotic products must contain the correct strains and the correct dose to achieve the claimed health benefits (Tripathi and Giri, 2014;Kolaček et al., 2017; Sánchez et al., 2017; Jackson et al., 2019). The ability to accurately identify and enumerate probiotic strains depend on the availability of reliable methods. Unfortunately, only a small percentage of probiotic strains available in the global market have reliable methods that enable strain level identification and enumeration. *L. paracasei* strain 8700:2 is a probiotic strain with diverse benefits to human health, yet no methods were developed for strain specific identification and enumeration. *L. paracasei* strain 8700:2 health benefits include reducing the severity of common cold symptoms (Lazou Ahrén et al., 2020), reducing the risk of acquiring common cold infections (Berggren et al., 2011; Regina et al., 2013), decreasing attack frequency in PFAPA syndrome patients (Batu et al., 2022), antagonistic activities against *Salmonella enterica* ssp. *enterica* and *Helicobacter pylori* (Hütt et al., 2006).

In this study, strain specific methods were developed and validated for the identification and enumeration of strain *L. paracasei* 8700:2 to support quality control and quality assurance efforts during probiotic product manufacturing and production. The methods can also be used when conducting laboratory or clinical research on strain *L. paracasei* 8700:2. Both the identification and enumeration methods are real-time PCR based methods.

Real-time PCR technique was selected since it brings many advantages when compared to the gold standard enumeration method, plate count methods. Real-time PCR based methods offer shorter time to results compared to plate count methods. Additionally, real-time PCR based methods are less labor intensive, can be strain-specific, highly sensitive, highly precise, and eliminate the need for specific growth media and specific incubation conditions (Hansen et al., 2018; Jackson et al., 2019). These advantages of real-time PCR based methods offer contemporary science-based advancements to the antiquated standard plate count methods that could save the industry considerable resources used for routine quality assurance testing. A major limitation to plate count methods is the inability to count VBNC cells (Kell et al., 1998; Lahtinen et al., 2006; Davis, 2014; Wilkinson, 2018; Gorsuch et al., 2019). VBNC cells have a low metabolic activity and are unable to divide before they undergo a resuscitation phase (Fiore et al., 2020), however, VBNC cells possess an intact cytoplasmic membrane as well as many typical properties of viable cells and maintain some metabolic activity. VBNC cells can reacquire their ability to reproduce under favorable environmental conditions of the gut, and to interact with the host and host’s microbiota as cultivable viable bacteria do (Fiore et al., 2020). This lead to questioning the ability of plate count methods to accurately count active cells in probiotic products when they do not take into account cells in VBNC state (Foglia et al., 2020). On the other hand, viability PCR based methods can enumerate VBNC cells, meaning that they are more accurate than plate count methods in determining viable counts.

To design a strain-specific assay for strain *L. paracasei* 8700:2, a target sequence that is unique to the target strain should be selected carefully from the genome sequence to achieve strain level specificity. The target sequence region for strain *L. paracasei* 8700:2 was selected using RAST system and the primers and probe were designed to amplify a 149 bp amplicon. The methods were validated for the qualitative identification, and then for the quantitative enumeration of strain *L. paracasei* 8700:2.

First, a method for the qualitative identification of strain *L. paracasei* 8700:2 was validated for specificity and sensitivity (Broeders et al., 2014; Shehata et al., 2019). Assay specificity (ability to correctly identify strain *L. paracasei* 8700:2 only) was evaluated *in silico*. The target sequence did not match any bacterial sequence on NCBI GenBank. Analytical specificity of the developed method was further confirmed experimentally using 10 target samples and 23 non-target strains including closely related strains such as *L. paracasei* and *L. casei* strains (HA-196, HA-274, R0215, R0422, HA-108, UALpc-04, Lpc-37, UALc-03, Lc-11). While all target samples amplified with mean Cq between 23.98 and 24.86, none of the non-target strains showed any amplification in real-time PCR, indicating strain level specificity (Table 1).

Assay sensitivity of the identification method for strain *L. paracasei* 8700:2 was also evaluated. The assay proved to be highly sensitive with a limit of detection of 0.5 pg. of DNA, corresponding to 150 copies. Assay sensitivity is the minimum amount of target that can be detected by an assay (Bustin et al., 2009). A highly sensitive assay enables the detection of small amounts of target strain’s DNA. This is important when dealing with difficult or complex sample matrix that gives low DNA yields, or when dealing with multi-strain blends containing the target strain at a low ratio.

Second, a method for the quantitative enumeration of strain *L. paracasei* 8700:2 was optimized for a viability treatment, validated for efficiency and precision, then its performance was evaluated compared to other enumeration methods (Bustin et al., 2009). This quantitative method was based on viability PCR to enable the quantification of viable cells only. In viability PCR, cells are treated with a DNA-intercalating dye such as propidium monoazide (PMA) or PMAxx to inactivate DNA from dead cells. The optimal viability dye concentration that can reliably inactivate DNA from dead cells varies depending on the target strain (Kiefer et al., 2020). Thus, it is important to optimize the viability treatment for each target strain. For example, the optimal final concentrations of PMA in previous studies ranged from 25 to 100 μM (Gobert et al., 2018; Hansen et al., 2018; Scariot et al., 2018; Shehata and Newmaster, 2021). A final concentration of PMAxx of 50 μM was chosen as the optimal concentration to inactivate DNA from dead cells of strain *L. paracasei* 8700:2 (Figure 2). Following viability treatment, DNA was liberated from cells using bead beating, since bead beating was reported to be effective in liberating DNA compared to other methods (Hansen et al., 2018), and commercial DNA extraction kits were avoided since they do not achieve 100% DNA recovery (Mumy and Findlay, 2004; Hansen et al., 2018). Bead beating for 5 min at 3,000 rpm was selected as it was previously found to be an effective technique for DNA liberation (Shehata and Newmaster, 2021).

The quantitative enumeration method of strain *L. paracasei* 8700:2 was then validated for efficiency. Reaction efficiency is very critical for a quantitative method. An ideal reaction efficiency of 100% means that all target molecules duplicate after every cycle. Reaction efficiency for a quantitative method is recommended to be between 90 and 110% with *R*^2^ values ≥0.98 (Broeders et al., 2014). Efficiency of the method developed for strain *L. paracasei* 8700:2 was 91.5, 93.7 and 90.9% with *R*^2^ values of 0.9984, 0.9928 and 0.9965 (Figure 3). Thus, the assay met the recommended efficiency and linearity parameters (Broeders et al., 2014). Additionally, the linear dynamic range of the assay was established between 6*10^2^ to 6*10^5^ genomes, covering 4 orders of magnitude (Figure 3). The dynamic range of an assay should span a minimum of three orders of magnitude and ideally should cover 5–6 dilutions (Bustin et al., 2009). Thus, the assay achieved an acceptable linear dynamic range.

The quantitative enumeration method of strain *L. paracasei* 8700:2 was then validated for precision by determining repeatability and reproducibility (Kralik and Ricchi, 2017). The RSD% for repeatability and for reproducibility was always below 5% (Figure 4). This value is well below the acceptable value of ≤ 25% for repeatability and reproducibility (Broeders et al., 2014). Thus, the assay proved to be precise with low technical variation.

The performance of the quantitative enumeration method of strain *L. paracasei* 8700:2 was evaluated compared to other enumeration methods (plate count and viability droplet digital PCR methods). When comparing viability real-time PCR methods to plate count method, the Pearson correlation coefficient (*r*) was 0.707 (Figure 5A). A previous study found a similar correlation (*r* = 0.76) between viability ddPCR and plate count methods for probiotic enumeration (Hansen et al., 2020). Method agreement analysis using Bland–Altman method comparison showed that viability real-time PCR always overestimated cell counts compared to plate count method (Relative difference ranging from 119 to 184%; Figure 6A). This may be explained by the presence of cells in VBNC state, which would by captured by viability real-time PCR but not using plate count method. In a previous study, the 95% limit of agreement of plate count versus viability PCR was −72 to 43% indicating that viability PCR tended to give slightly higher counts compared to plate count method (Hansen et al., 2020).

On the other hand, when comparing viability real-time PCR methods to viability ddPCR, the Pearson correlation coefficient (*r*) was 0.922 (Figure 5B). Method agreement analysis using Bland–Altman method comparison showed that there was general agreement between the two methods (Relative difference ranging from −20 to 22%; Figure 6B). This may be explained by the ability of both methods to detect VBNC cells. Previous studies that compared real-time PCR and ddPCR for quantification of bacteria and viruses reported general agreement, overestimation, or underestimation by real-time PCR (Gosselin-Théberge et al., 2016; Verhaegen et al., 2016; Witte et al., 2016; Papić et al., 2017; Ricchi et al., 2017).

Assessing the effect of heat treatments on viable counts determined using *L. paracasei* 8700:2 viability real-time PCR and plate count methods showed that heating at 60°C, or higher resulted in no detectable viable cells using both methods. This is potentially caused by complete cell death by heating at ≥ 60°C. Heating at 50°C and at 40°C resulted in lower viable counts compared to non-heated cells using both methods, however, the reduction was higher with plate count methods compared to viability real-time PCR (9-fold and 5-fold at 50°C, and 3- and 2-fold at 40°C, respectively; Table 3). The difference can be possibly explained by the portion of cells that enter a VBNC state after heating. Unfavorable conditions such as high temperature can induce cells to enter a VBNC state, which can be captured by viability real-time PCR but not using plate count method. When assessing the ability of *L. paracasei* 8700:2 viability real-time PCR method to detect cell death following storage, it was found that both methods detected cell death after storage for 1 year, shown as 2.2- to 4.4-fold reduction using plate count method and 1.5- to 2.4-fold reduction using viability real-time PCR (Figure 7). Probiotic strain viability is known to be affected by storage conditions such as temperature, moisture, and packaging type (Tripathi and Giri, 2014), and poor strain stability during shelf life of probiotic products is one of the major challenges facing the probiotic industry (Morovic et al., 2016). The results show that a slightly higher reduction in cell counts was observed with plate count method after storage for 1 year. Plate count methods rely on cultivability while viability PCR rely on membrane integrity to determine viability, and cell cultivability declines faster than membrane integrity (Foglia et al., 2020). Thus, the higher count reduction observed with plate count can potentially be explained by the inability of plate count method to detect cells in VBNC state, the ratio of which is expected to increase during storage. A previous study reported that pate count method showed a higher reduction compared to flow cytometry following storage, which was explained by a dynamic shift of bacterial cells into VBNC or dormant state during storage (Foglia et al., 2020). Since VBNC cells are considered probiotics, cell counts determined using viability real-time PCR would be more accurate in determining viable counts. Viability real-time PCR are particularly valuable when enumerating probiotics following storage and during shelf life of finished products.

## Conclusion

The overall results show that the developed method for the identification of *L. paracasei* 8700:2 is strain specific, sensitive and allows for fast, simple and cost-effective identification of strain *L. paracasei* 8700:2. Furthermore, the method developed for the enumeration of *L. paracasei* 8700:2 is sensitive, efficient, precise and provide a significantly shorter time to results compared to plate count methods, which can save considerable resources in routine quality assurance testing. The method also enables the specific enumeration of *L. paracasei* 8700:2 in mono-strain or multi-strain samples and enables the enumeration of cells in VBNC state, which cannot be achieved using plate count methods. Thus, the identification and enumeration methods developed in this study can support higher quality assurance measures in the probiotic industry with considerable cost savings.

## Data availability statement

The original contributions presented in the study are included in the article/supplementary material, further inquiries can be directed to the corresponding author.

## Author contributions

HS designed the study, analyzed the data, and wrote the manuscript. HS and BH carried out the experiments. SN helped to design the study, facilitated sample acquisition, and edited the manuscript. All authors contributed to the article and approved the submitted version.

## Funding

This study was supported by the Natural Health Product Research Alliance (NHPRA), University of Guelph.

## Conflict of interest

HS and BH were employed by Purity-IQ Inc.

The remaining author declares that the research was conducted in the absence of any commercial or financial relationships that could be construed as a potential conflict of interest.

## Publisher’s note

All claims expressed in this article are solely those of the authors and do not necessarily represent those of their affiliated organizations, or those of the publisher, the editors and the reviewers. Any product that may be evaluated in this article, or claim that may be made by its manufacturer, is not guaranteed or endorsed by the publisher.
